# Citrus Huanglongbing Detection Based on Multi-Modal Feature Fusion Learning

**DOI:** 10.3389/fpls.2021.809506

**Published:** 2021-12-23

**Authors:** Dongzi Yang, Fengcheng Wang, Yuqi Hu, Yubin Lan, Xiaoling Deng

**Affiliations:** ^1^College of Electronic Engineering, College of Artificial Intelligence, South China Agricultural University, Guangzhou, China; ^2^National Center for International Collaboration Research on Precision Agricultural Aviation Pesticide Spraying Technology, Guangzhou, China; ^3^Guangdong Laboratory for Lingnan Modern Agriculture, Guangzhou, China; ^4^Guangdong Engineering Technology Research Center of Smart Agriculture, Guangzhou, China

**Keywords:** convolutional neural network, citrus greening disease, machine learning, multi-modal feature fusion, hyperspectral images

## Abstract

Citrus Huanglongbing (HLB), also named citrus greening disease, occurs worldwide and is known as a citrus cancer without an effective treatment. The symptoms of HLB are similar to those of nutritional deficiency or other disease. The methods based on single-source information, such as RGB images or hyperspectral data, are not able to achieve great detection performance. In this study, a multi-modal feature fusion network, combining a RGB image network and hyperspectral band extraction network, was proposed to recognize HLB from four categories (HLB, suspected HLB, Zn-deficient, and healthy). Three contributions including a dimension-reduction scheme for hyperspectral data based on a soft attention mechanism, a feature fusion proposal based on a bilinear fusion method, and auxiliary classifiers to extract more useful information are introduced in this manuscript. The multi-modal feature fusion network can effectively classify the above four types of citrus leaves and is better than single-modal classifiers. In experiments, the highest accuracy of multi-modal network recognition was 97.89% when the amount of data was not very abundant (1,325 images of the four aforementioned types and 1,325 pieces of hyperspectral data), while the single-modal network with RGB images only achieved 87.98% recognition and the single-modal network using hyperspectral information only 89%. Results show that the proposed multi-modal network implementing the concept of multi-source information fusion provides a better way to detect citrus HLB and citrus deficiency.

## Introduction

Citrus Huanglongbing (HLB), also called citrus greening, is commonly believed to be citrus cancer without effective treatment. The symptoms of HLB are mainly yellow shoots, yellow leaves, and red nose fruits, among others. The infected plants easily wither and die. HLB is found all over the World, and it also occurs in China, especially in the Guangdong Sihui and Guangdong Huizhou. HLB is infectious and can be spread through insect vectors or grafting. The three most effective methods to prevent HLB are planting non-toxic seedlings, preventing and controlling citrus psyllids, and removing diseased plants (Han et al., 2021). In traditional agriculture, the prevention and control of HLB relies on the observation of experts or experienced farmers to remove diseased plants as early as possible. For plants with mild symptoms, PCR (Polymerase Chain Reaction), and other biotechnological techniques can be used to accurately identify plants. This method has high accuracy and disease can be detected and eradicated in the early stages of plant infection. However, this approach relies on experts first identifying diseased plants, and then bringing the diseased plants back to the laboratory to have disease confirmed by genetic methods. This process is lengthy and dependent on those experts. If a machine is trained as an expert and replaces the expert for identification, the detection process will be significantly accelerated.

With the development of deep learning since 2015, many useful networks for special object extraction have emerged, such as CNNs,ResNet50 (He et al., 2016), VGG16 (Simonyan and Zisserman, 2014), GoogleNet (Szegedy et al., 2015), SeNet50 (Hu et al., 2020), ResNeXt101 (Szegedy et al., 2015), VGG (Simonyan and Zisserman, 2014), and Senet50 (Hu et al., 2020). They have been very successful in modeling complicated systems, owing to their ability of distinguishing patterns and extracting regularities from data. The above-mentioned networks have been effectively incorporated in plant phenotyping projects. For example, variety identification in seeds (Taheri-Garavand et al., 2021b; Plants 10, 1406) and in intact plants by using leaves (Nasiri et al., 2021; Plants 10, 1628), weed and crop classification and recognition is the frontier and trend of agricultural artificial intelligence (Deng et al., 2020; Jiang et al., 2020), detecting crop nutritional deficiencies (Baresel et al., 2017; Tao et al., 2020), and plant disease classification (Kaya et al., 2019; Karlekar and Seal, 2020). Mostly, studies learn single-source information, and classify or identify subsequent information. These kinds of networks mostly use visual image and have rather good accuracy in specific cases. However, agriculture is a complicated system in which the shooting conditions of visual images randomly change and the crops keep growing, which leads the networks reliant on visual imaging to lack universality. Several researchers have made some efforts to improve the accuracy by continuously supplementing datasets (Picon et al., 2019), yet data collecting is a very tough work in agriculture as it is restricted by the environment and the growth cycle of plants. Therefore, how to improve the precision rate under unabundant dataset is becoming increasingly more significant.

In recent years, with the rapid development of spectroscopy, some studies adopted multispectral and hyperspectral information to detect deeper information of objects, such as using infrared to evaluate the quality of strawberry by hyperspectral images (Su et al., 2021), using hyperspectral satellite remote sensing to estimate grassland yield (Ali et al., 2014), or using UVA-based hyperspectral imagery (Feng et al., 2020) for yield prediction. Compared with RGB images, hyperspectral images combined with neural network technology can more effectively identify plant diseases, even in the early stage of disease.

The internal information extracted from hyperspectral images can be used to compensate for the shortcomings of RGB images with only surface information. Hence, multi-source feature fusion can improve the predictive ability of the model. The purpose of the fusion model is to combine the strengths of different sub-models to compensate for any shortcomings (Zadeh et al., 2017). Deep multi-modal learning can reduce the design requirements for feature engineering and deep-learning architectures, and can achieve the required accuracy more simply and quickly (Atrey et al., 2010; Ramachandram and Taylor, 2017; Baltrusaitis et al., 2019). Yan et al. (2021) proposed a fusion scheme combining a multi-dimensional convolutional neural network with a visualization method for detection of aphis gossypii glover infection in cotton leaves using hyperspectral imaging, which achieved good development prospects in plant disease identification.

Numerous researchers have conducted laboratory investigations into the identification of HLB using different methods under different observation heights, such as using visual images in the laboratory with traditional machine-learning methods (Deng et al., 2016) and using UAV hyperspectral and multispectral images using deep-learning networks (Deng et al., 2019; Lan et al., 2020).

To increase the reliability and precision of HLB detection, in this study, a method is proposed that fuses two sources of information, namely, spectral and RGB images, by building a multi-modal deep-learning network to identify HLB leaves from four categories.

## Materials and Methods

### Data Acquisition and Processing

**FIGURE 1:**
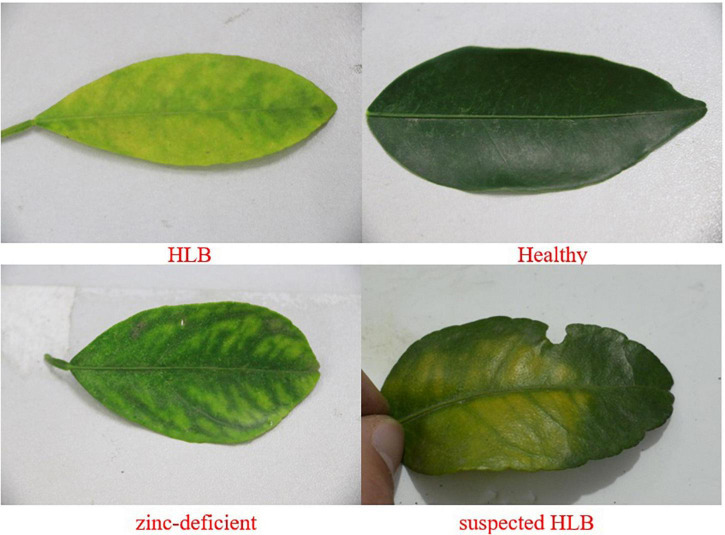
Dataset collection location.

**FIGURE 2:**
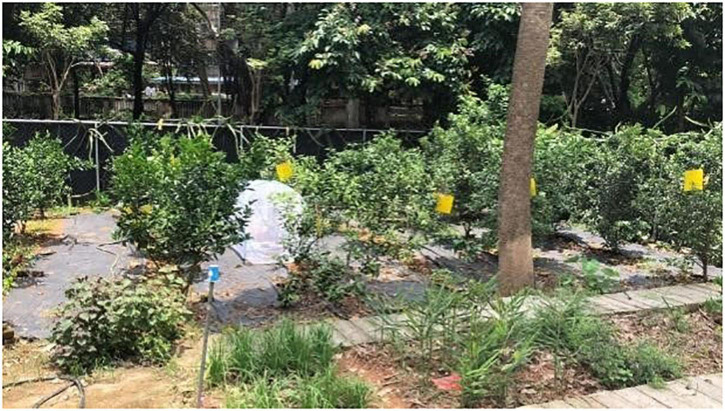
Four categories leaves.

The data used in this study were collected in the citrus test fruit field of South China Agricultural University, Tianhe, Guangdong Province (longitude 113.35875, latitude 23.15747). In early March, citrus trees are in the spring growth period and are grown in subtropical climate regions, shown in Figure 1. The variety of citrus is Shatangju (*Citrus reticulata Banco*). The selected tree samples were specially cultivated and PCR-tested, and Zinc deficiency was visually assessed by a field expert, and was confirmed by conducting mineral analysis. The data samples of this study include the leaves of HLB plants, of Zn-deficient plants, of healthy plants, and those with suspected HLB (in which case the surface of the leaf is uniformly yellow, which is different from the obvious symptoms of HLB). The four categories leaves are shown in Figure 2.

**FIGURE 3:**
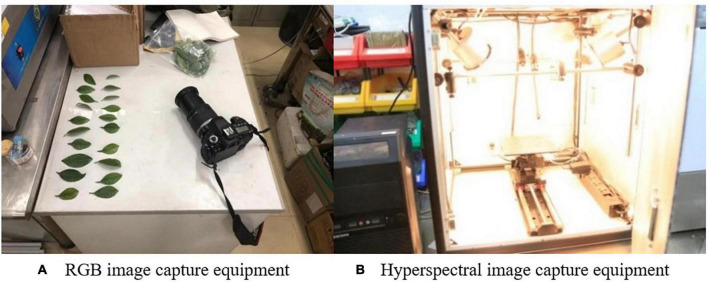
Data collection equipment. **(A)** RGB image capture equipment. **(B)** Hyperspectral image capture equipment.

The collection environment is shown in Figure 3. The RGB images were taken with Sony cameras and under natural light, ensuring that the required foliage was clear, independent of the shooting location, and free of background interference. The distance between camera and leaves was controlled with 20–50 cm. The hyperspectral data of leaves were collected by a hyperspectral imager (Hypersis-VNIR-PFH, Zhuoli Hanguang, Beijing, China). The spectral range was 300 nm to 1070 nm and the exposure time for each collection was 30 ms. The running speed of the mobile platform was 5.0375 mm/s, the scanning distance 120 mm, and the hyperspectral image size 100 × 200 pixels. Spectral data analysis and processing were implemented in ENVI 5.3 software (Harris Geospatial Solutions, Inc., Broomfield, CO, United States).

**FIGURE 4:**
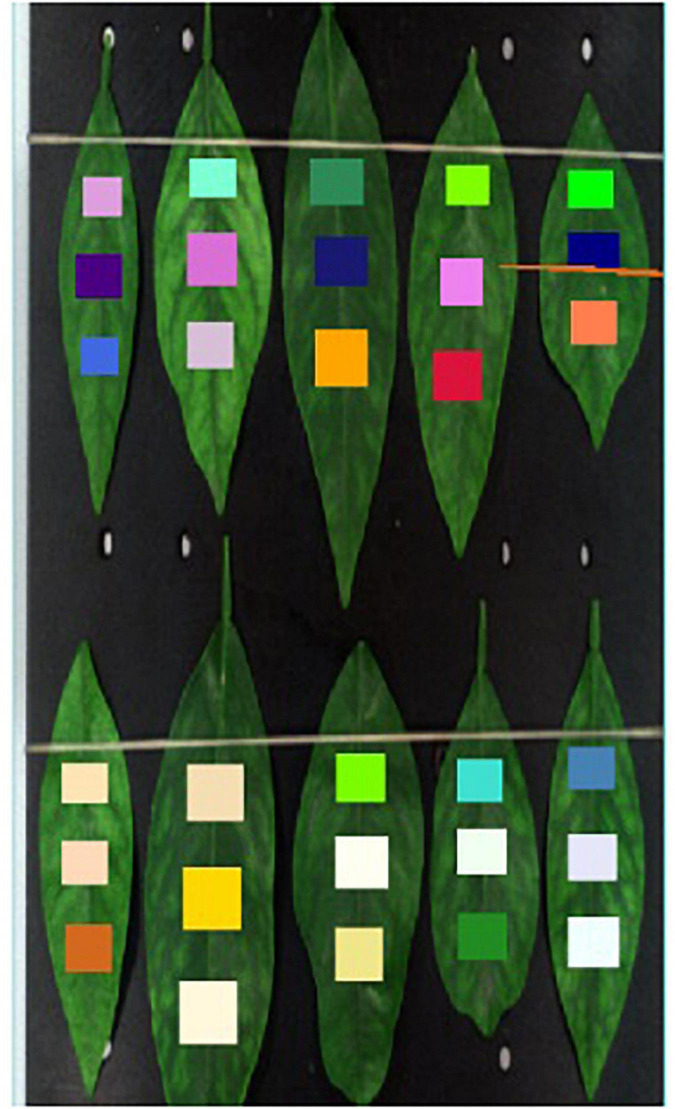
Feature area selection during processing in Envi software.

Figure 4 shows the method of feature area selection during the data processing step. In the process of hyperspectral image analysis, the upper, middle, and lower regions of interest of the leaf blade were chosen as the feature region, the average reflectance in the region of interest calculated, and the average reflectance used to represent the area. Finally, the hyperspectral image was converted into a hyperspectral band, and the average reflectance used to reflect the area. The frequency band of each area ranged from 300 nm to 1070 nm, removing the incomplete information about the start and the tail, leaving 768 bands in the middle. Owing to the similarity of adjacent bands of hyperspectral images, to reduce similar repetitive features, every three adjacent bands in the 300–1070-nm range were extracted and combined into a new band. After final extraction, 256 composite bands remained.

Table 1 shows the one-to-one correspondence dataset between images and spectral data. Each RGB image corresponds to a spectral sample and each piece of spectral data contains the spectral information of the upper, middle, and lower regions of the leaf.

**TABLE 1:** Four different types of data and amounts of each.

Species	Number of images	Number of spectral images
Healthy HLB	300 375	300 375
Zn-deficient HLB suspected	350 300	350 300

*HLB, Citrus Huanglongbing.*

### Multi-Modal Network Architecture

**FIGURE 5:**
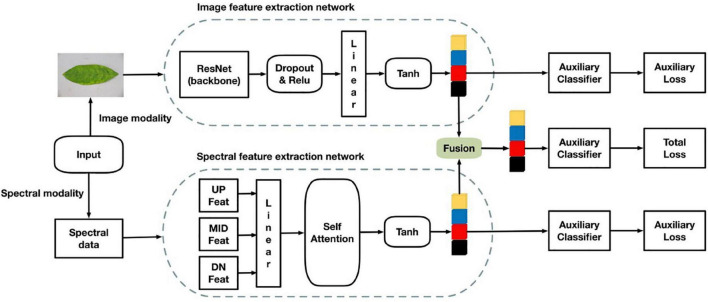
Multi-modal network structure.

The multi-modal network proposed in this study consists of two backbone networks. The architecture was divided into four parts. The first is an image feature extraction network that extracts surface features of RGB images. The second is a hyperspectral band feature extraction network that extracts the HLB feature bands. The third is a feature fusion part that fuses the two features extracted from two different networks and performs classification with an auxiliary classifier. The fourth part is classification using auxiliary classifiers. The multi-modal network structure is shown in Figure 5.

### RGB Image Extraction Network

In the first part of RGB image feature extraction, ResNet50, VGG16, and ResNeXt101 were selected as the candidates for the backbone network. After experimental comparison, ResNet50 was adopted because it works well and in wide use. In terms of the network structure, ResNet50 has fewer parameters, but the effect achieved is similar to that of ResNeXt101. The image in this experiment is high definition, and the amount of calculation required for the extraction of the hyperspectral band is also large. To reduce the amount of calculation and not lose too much accuracy, ResNet50 was chosen. The results of the experiment are detailed further below at Table 2. To enrich the diversity of samples, a data enhancement module was added to the network. During the training process, there was a 10% probability that the RGB image would be randomly rotated forward or counterclockwise by 45°. The feature dimensionality extracted from the backbone network was 2048. To reduce the dimensionality obtained by feature fusion and reduce the amount of network calculation, the fully connected layer was used for feature dimensionality reduction, and the final image feature dimensionality obtained was 256.

**TABLE 2:** Single-network classification and multi-modal network classification accuracy.

Sample	Model	Accuracy (%)
RGB image hyperspectral data	ResNet50 VGG16 ResNeXt101 Hyperspectral feature extraction network	85 84.51 87.98 89
RGB image + hyperspectral data	Multi-modal network M1 Multi-modal network M2 Multi-modal network M3	96 95.1 97.89

*M1, ResNet50+hyperspectral feature extraction network; M2, VGG16+hyperspectral feature extraction network; M3, ResNeXt101+hyperspectral feature extraction network.

### Feature Extraction Network for Hyperspectral Band

**FIGURE 6:**
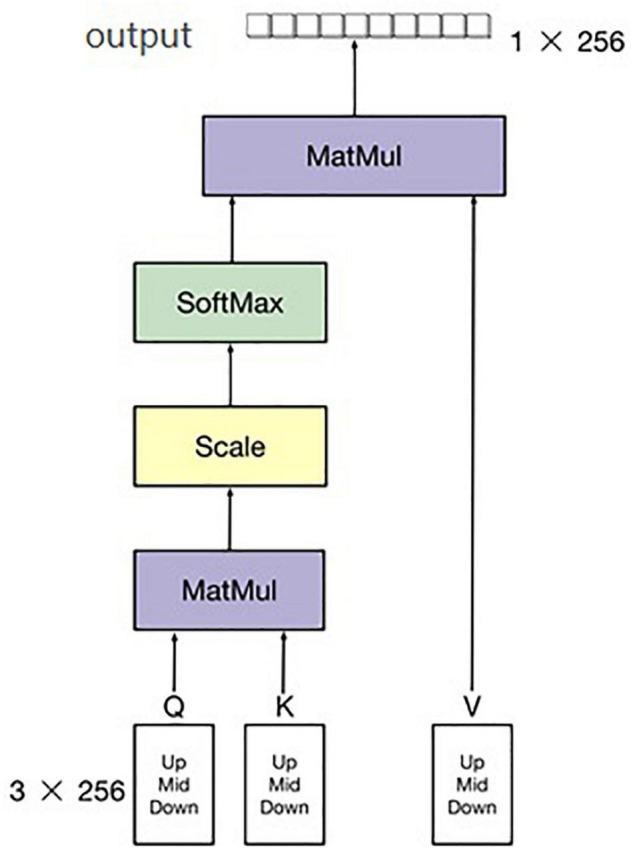
Hyperspectral band feature extraction network.

The second part of the multi-modal network is to extract feature band information of hyperspectral data. There are many common spectral feature band extraction methods, such as support vector machines and PCA (Principal Component Analysis), among others (Velasco-Forero and Angulo, 2013; Deng et al., 2014; Medjahed et al., 2015; Pérez et al., 2016). In this study, a simple neural network for feature extraction among the 300–1070-nm hyperspectral data is proposed, and an attention module was added in this hyperspectral feature band exaction network to increase the ability of extracting bands. After combining the three adjacent bands into one channel, the number of bands decreased from 758 to 256, which reduced the overall amount of calculation and number of parameters of the hyperspectral feature extraction network. Hyperspectral band information is one-dimensional (1D) information. Commonly used image neural networks are not suitable for 1D information extraction, and we only needed to extract the bands with large differences. Therefore, the designed neural network must be capable of 1D information extraction. Moreover, it must be able to find the bands with large differences and retain the characteristic of this large difference. As shown in Figure 4, the upper, middle, and lower parts of each hyperspectral image were selected, and the hyperspectral band of each hyperspectral image calculated by averaging each part of the sample. Thus, there were three pieces of hyperspectral 1D data for each channel of the hyperspectral image. Therefore, the input of the hyperspectral band feature extraction network was 256 × 3. Even so, a significant amount of redundant information remains. To reduce the influence of this redundant information on the final classification results, a soft attention mechanism was adopted in the module to further extract the hyperspectral information of input data. Finally, the output size of the network was 1 × 256. The structure of the attention algorithm is shown in Figure 6.

### Multi-Modal Feature Fusion

**FIGURE 7:**
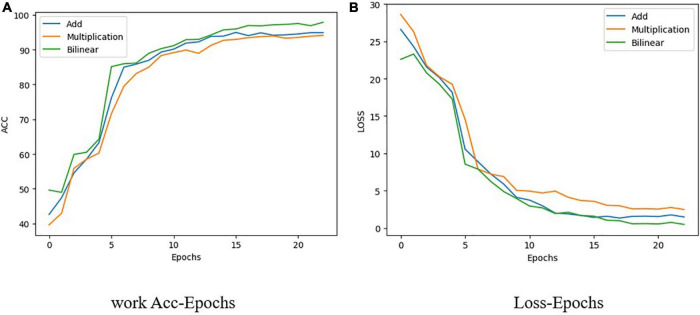
Change with epoch of loss and accuracy of three feature fusion methods used in present. **(A)** Work Acc-Epochs. **(B)** Loss-Epochs.

Typical fusion methods mainly comprise early and late fusion. As the name suggests, early fusion is used to fuse features at feature levels, using operations such as concatenation and addition of different features (Chaib et al., 2017), and then inputting the fused features into a model for training. Late fusion refers to fusion on the score level. Methods such as a feature pyramid network (Pan et al., 2019) train multiple models, and each model will have a prediction score. The results of all models are fused to obtain the final prediction results. In this study, the 1D hyperspectral band information and 3D RGB picture information were fused before detection. ResNet50 and a hyperspectral band feature extraction network (spectrum) were used in the present work as the fusion network to carry out three different feature fusions, all of which are examples of early fusion. These three methods are feature addition, feature multiplication, and feature bilinear fusion. From Figure 7 shows that the accuracy of addition is 94.58%, that of multiplication is 93.85%, and that of bilinear fusion is 95.1%. It can also be seen from Figure 7 that the fitting speed of bilinear fusion was also faster than that of the other two methods.

The bilinear fusion method (Yu et al., 2018) was adopted to fuse the features between different networks. The original bilinear fusion is shown in Eqs. (1) and (2). The two input modes are **X** and **Y**, and the bilinear fusion can thus be expressed as:


(1) $${\text{Z}}_{i}={\text{X}}^{T}\,{\text{W}}_{i}\,\text{Y},$$

where, **W** is the projection matrix and **Z** the output of the bilinear model. **W** is decomposed into two low-rank **U** and **V** matrices, with ° indicating a matrix dot product:


(2) $${\text{Z}}_{i}={\text{X}}^{T}\,{\text{U}}_{i}\,{\text{V}}_{i}^{T}\,\text{Y}={\text{U}}_{i}^{T}\,{\text{X}}^{{}^{\circ}}\,{\text{V}}_{I}^{T}\,\text{Y}.$$

The specific fusion formula is shown in Equation (3), where Z(*Features*_*Mix*_) represents the fusion features, I (*Features*_*Image*_) the features extracted by the image network, and B (*Features*_*Spectrum*_) the features extracted by the spectral network. A is an N × N matrix and Bias an N × 1 matrix; in the experiments detailed herein, *N* = 256.


(3) $$\text{Z}({\text{Features}}_{\mathrm{mix})}=\text{I}({\text{Features}}_{\mathrm{Image})}\text{A B}({\text{Features}}_{\mathrm{band})}+\text{Bias}$$

### Auxiliary Classifier

**FIGURE 8:**
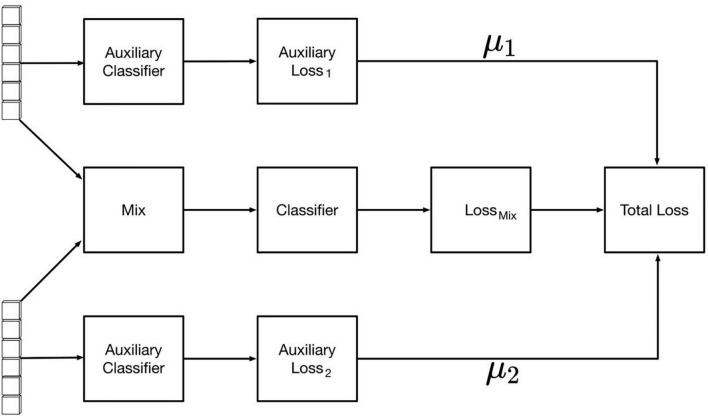
Loss calculation method based on auxiliary and mixture loss.


After feature fusion, the samples were modeled using auxiliary classifier based on the fused feature values. The final classification effect of the network is affected by the two backbone feature extraction networks. To improve the feature extraction effects of the RGB image feature extraction network and hyperspectral band feature extraction network, the auxiliary classifiers were modified as shown in Figure 8, where the loss of the overall network consists of the loss of the fusion feature classifier and one of each backbone network classifier. The specific loss calculation formula is as in Equation (2), where *TotalLoss* represents the overall loss value of the network, **Loss_mix_** the loss value of the fusion feature classifier, **Aux** **Loss_1_** the loss value of the image auxiliary classifier, and **AuxLoss_2_** the spectral auxiliary classifier The loss values of μ**_1_** and μ_2_ are the auxiliary classifier loss weight coefficients (0 ≤μ**_1_** < 1,0 ≤ μ**_2_** < 1). By testing different groups of weight coefficient values, it was found that the best classification effect is obtained when the coefficient μ**_1_** = 0.25 and the coefficient μ_2_ = 0.20.

(4) $$\text{Total}\;\text{Loss}={\text{Loss}}_{\mathrm{mix}}+\mu_{1}\times \text{Aux}\;{\text{Loss}}_{1}+\mu_{2}\times \text{Aux}\;{\text{Loss}}_{2}$$

## Results

The experimental hardware environment of this study is listed in Table 3. The software environment was set as the following: python, Ubuntu 16.04, CUDA, CUDNN, and OpenCV. In this study, **F1** score and accuracy were used to evaluate the trained model. The formulas are given in Equations (3)–(6), where **P** is the precision rate, **R** the recall rate, **TP** the number of true positive samples, **FP** the number of false positive samples, **FN** the number of false negative samples, **true**_**num** the number of samples that are classified correctly, and **total**_**num** the total number of tests and total number of samples.

**TABLE 3:** Experimental environment.

Hardware	Brand	Number
CPU Storage Graphics card Hard disk	I7–10700 Kingston, 16 GB GeForce GTX3070 West Statistics, 1 TB	1 2 1 1
Main board	Dell Precision 3640 tower	1


(5) $$\text{P}=\text{P}=\frac{\text{TP}}{\text{TP}+\text{FP}}$$

(6) $$\text{R}=\frac{\text{TP}}{\text{TP}+\text{FN}}$$

(7) $$\text{F1}=\frac{2\,\text{P}*\text{R}}{\text{P}+\text{R}}$$

(8) $$\text{Accuracy}=\frac{\text{true}\,\_\,\text{num}}{\text{total}\,\_\,\text{num}}$$

### Experimental Results

The experimental comparison results between single-network and multi-modal network classification are shown in Table 2. The recognition accuracies of the single network using RGB images were 85, 84.51, and 87.98% based on ResNet50, VGG16, and ResNeXt101, respectively. The recognition accuracy of the hyperspectral data dimensionality reduction network based on the soft attention mechanism was 89%, while that of the multi-modal networks designated M1 (ResNet50+hyperspectral feature extraction network), that designated M2 (VGG16+hyperspectral feature extraction network), and that designated M3 (ResNext101 +hyperspectral feature extraction network) reached 96, 95.1, and 98% respectively, all significantly higher than that of a single network. Compared with the F1 score of 85% using only the image network and that of 89% using only the spectral network, increases of 13 and 9%, respectively, were found. It can be clearly seen that feature fusion based on the bilinear fusion method and the multi-modal network of the auxiliary classifier can extract more useful information, and can better classify items with similar features.

To verify the performance of the multi-modal networks, ResNet50 with medium recognition accuracy (Table 2) was selected as the basic network to better reflect the improvement of recognition accuracy of multi-modal networks. Table 4 shows the detection performance of each category based on multi-modal network M1, where the F1 scores of HLB, healthy, Zn-deficient, and suspected HLB-diseased leaves reached 95, 98, 96, and 94%, respectively, showing that average recognition accuracy reached over 95%.

**TABLE 4:** Four classification results of multi-modal network M1.

Type	Precision (%)	Recall (%)	F1 score (%)
HLB	96	94	95
Health Zn-deficient HLB suspected	98 97 92	99 94 96	98 96 94

*M1, ResNet50+ hyperspectral feature extraction network. HLB, Citrus Huanglongbing.

### Visualization Analysis of Models

**FIGURE 9:**
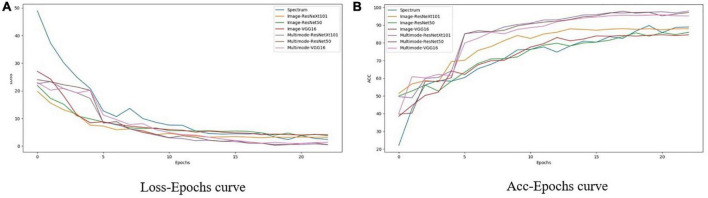
Change with epoch of loss and accuracy of different networks in training process. **(A)** Loss-Epochs curve. **(B)** Acc-Epochs curve.

Figure 9 shows the change of loss and accuracy with epoch during the training process of each network. It can be seen from Figure 9 that with increasing epoch loss, the fitting effect of the multi-modal model is obviously better than that of the RGB image network, and both tend to stabilize after 20 epochs. Compared with single-modal networks, including the spectrum network and RGB image networks using VGG16, ResNet50, and ResNeXt101, the three multi-modal networks achieved significantly better performance with faster convergence (as shown in Figure 9A) and higher accuracy (as shown in Figure 9B).

**FIGURE 10:**
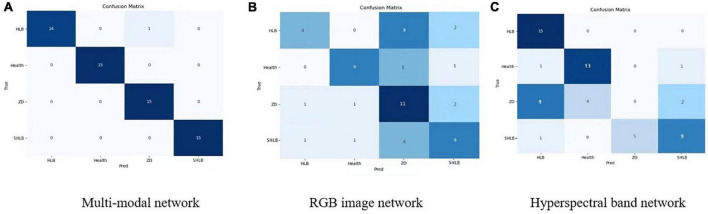
Confusion matrix of the three models. **(A)** Multi-modal network. **(B)** RGB image network. **(C)** Hyperspectral band network.

Figure 10 shows the confusion matrix of the three models. Figures 10B,C is the confusion matrix of the RGB image network and the hyperspectral network. It can be seen that the classification effects of the RGB image network and the hyperspectral image network have complementary aspects, especially for zinc deficiency. Classification of symptoms and HLB symptoms. Figure 10A is the effect of the final multi-modal network. It can be seen that the final confusion matrix has achieved a good effect.

## Discussion

Most existing networks can significantly improve the recognition accuracy by increasing the depth of the network, the dimensionality of the network, and the size of the data set. Such as ResNet, from ResNet50 to ResNet101, its recognition accuracy is improved, but the recognition speed and calculation amount are increased. When more than 101 layers are added, the recognition accuracy is not improved. This shows that although only increasing network depth can increase the accuracy, the cost is too high. The GoogleNet is to increase the width of each layer without increasing the depth of the network, but this improvement is also limited. Besides, dataset is difficult work in agriculture as it is restricted by the environment and the growth cycle of plants. Multi-modal networks can expand the data dimension through network fusion and fusion of features extracted from different data. Under the condition of insufficient data for a deep-learning network, it is relatively simple to combine other sources of information to improve the accuracy of the network from a horizontal perspective rather than a vertical perspective.

In the present study, the four testing categories discussed have similar symptoms, and are difficult to discriminate only by visual imaging. Hyperspectral data can reflect the internal information of plants to a certain extent, such as chlorophyll or element content, and can make up for the lack of RGB imagery and solve the discrimination problem resulting from the similar appearance of leaves.

Regarding the multi-feature fusion part, fusion weight coefficients were introduced to the weigh the output result, thus improving the fitting effect of the proposed multi-modal network. The image recognition accuracy of the multi-modal model can be even improved by adding more dimensional information or improving the performance of the constituent network. The proposed method can also be applied to other agricultural applications, such as pest and disease detection with similar symptoms or appearances.

On a commercial scale, evidently, a capital investment is initially required for adopting the employed approach (Taheri-Garavand et al., 2021a Industrial Crop Prod 171, 113985). Nevertheless, the wide-ranging large-scale commercial applications can provide high returns through considerable improvements in process enhancement and cost reduction. Spectroscopy is a high-cost and high-tech imaging device, and its application areas are still being developed. However, through the research in this article, it can further expand its application fields and improve its technology. Through the neural network fusion method and the combination of RGB images, the recognition and classification of agricultural pests or agricultural diseases are enhanced.

## Conclusion

A multi-modal network for citrus HLB detection and a bilinear fusion method based on RGB images and hyperspectral information are proposed in this study. Four HLB types with similar symptoms of leaves (HLB, suspected HLB, Zn-deficient, and healthy) were tested experimentally to verify the effectiveness of the multi-modal network. Results show that the F1-score of HLB detection based on multi-modal network reached 95%, that of healthy leaves reached 98%, that of Zn-deficient leaves reached 96 %, and that of suspected HLB diseased leaves reached 94%. The image recognition accuracy of the multi-modal model can effectively improve the recognition accuracy of the model when the size of the dataset is limited.

## Data Availability Statement

The raw data supporting the conclusions of this article will be made available by the authors, without undue reservation.

## Author Contributions

DY conceptualized the experiment, selected the algorithms, collected and analyzed the data, and wrote the manuscript. FW and YH trained the algorithms, collected and analyzed data, and wrote the manuscript. XD and YL supervised the project. All authors discussed and revised the manuscript.

## Conflict of Interest

The authors declare that the research was conducted in the absence of any commercial or financial relationships that could be construed as a potential conflict of interest.

## Publisher’s Note

All claims expressed in this article are solely those of the authors and do not necessarily represent those of their affiliated organizations, or those of the publisher, the editors and the reviewers. Any product that may be evaluated in this article, or claim that may be made by its manufacturer, is not guaranteed or endorsed by the publisher.

## Abbreviations

HLB: Citrus Huanglongbing
PCA: principal component analysis
PCR: polymerase chain reaction
UVA: unmanned aerial drone.
